# Heat Shock Factor 1 Depletion Sensitizes A172 Glioblastoma Cells to Temozolomide via Suppression of Cancer Stem Cell-Like Properties

**DOI:** 10.3390/ijms18020468

**Published:** 2017-02-22

**Authors:** Chang-Nim Im, Hye Hyeon Yun, Jeong-Hwa Lee

**Affiliations:** 1 Department of Biochemistry, The Catholic University of Korea, Seoul 06591, Korea; nice1205@hanmail.net; 2 Institute for Aging and Metabolic Diseases, The Catholic University of Korea, Seoul 06591, Korea; 3 Cancer Evolution Research Center, College of Medicine, The Catholic University of Korea, Seoul 06591, Korea

**Keywords:** BIS, HSF1, glioblastoma, temozolomide, apoptosis

## Abstract

Heat shock factor 1 (HSF1), a transcription factor activated by various stressors, regulates proliferation and apoptosis by inducing expression of target genes, such as heat shock proteins and Bcl-2 (B-cell lymphoma 2) interacting cell death suppressor (BIS). HSF1 also directly interacts with BIS, although it is still unclear whether this interaction is critical in the regulation of glioblastoma stem cells (GSCs). In this study, we examined whether small interfering RNA-mediated BIS knockdown decreased protein levels of HSF1 and subsequent nuclear localization under GSC-like sphere (SP)-forming conditions. Consistent with BIS depletion, HSF1 knockdown also reduced sex determining region Y (SRY)-box 2 (SOX2) expression, a marker of stemness, accompanying the decrease in SP-forming ability and matrix metalloprotease 2 (MMP2) activity. When HSF1 or BIS knockdown was combined with temozolomide (TMZ) treatment, a standard drug used in glioblastoma therapy, apoptosis increased, as measured by an increase in poly (ADP-ribose) polymerase (PARP) cleavage, whereas cancer stem-like properties, such as colony-forming activity and SOX2 protein expression, decreased. Taken together, our findings suggest that targeting BIS or HSF1 could be a viable therapeutic strategy for GSCs resistant to conventional TMZ treatment.

## 1. Introduction

Glioblastoma multiforme (GBM) is the most aggressive of brain tumors. Glioblastoma stem cells (GSCs) are a subpopulation of tumor cells that develop resistance to the DNA alkylating agent temozolomide (TMZ), the conventional drug used in GBM therapy following surgery, resulting in tumor recurrence [1]. Targeting GSCs has recently been considered a potential strategy, as the development of resistance limits its therapeutic applicability, similar to many other anti-cancer therapies.

Heat shock factor 1 (HSF1) is an important regulator of protein quality control via the induction of heat shock proteins (HSPs) [2]. HSF1 is considered a potential target for anti-cancer therapy because it is overexpressed in several cancers, including breast cancer, and increased expression has been associated with poor prognosis [3,4]. In addition, there is increasing evidence indicating that HSF1 is involved in the cancer stem cell (CSC)-like phenotype. Wang et al. demonstrated that HSF1 overexpression promoted and HSF1 knockdown inhibited the CSC-like phenotype without affecting expression of HSP70 and HSP90 in several breast cancer cell lines, including MCF7 and T47D, suggesting that HSF1 regulated the CSC-like phenotype by targeting non-*HSP* genes [5].

Bcl-2 (B-cell lymphoma 2) interacting cell death suppressor (BIS) [6], a member of the Bcl-2-associated anthanogene co-chaperone family, is an HSF1 target gene that promotes cell survival in normal and neoplastic cell types via its interaction with a variety of partners, including HSP70, Bcl-2, and others [7]. Transcription of BIS is regulated by HSF1 through two main heat shock elements (HSEs) in the BIS promoter region in response to a variety of stimuli, including hydrogen peroxide and the proteasome inhibitor MG132, indicating that the transcription factor plays an important role in *BIS* gene expression [8,9,10]. HSF1-dependent expression of *BIS* has been shown to reduce apoptosis in 4-hydroxynonenal-treated colon cancer cells [11]. BIS also interacts with HSF1 [12] and translocates to the nucleus. Depletion of BIS reduced nuclear HSF1 [13] and phosphorylated BIS altered HSF1 translocation resulting in HSP70 promoter activity [14], suggesting that reciprocal regulation of HSF1 and BIS occurs under conditions of oxidative stress, such as heat shock and hydrogen peroxide treatment.

In a previous study, we determined specific conditions in which the formation of GSC-like spheres (SPs) were enriched with increased sex determining region Y (SRY)-box 2 (SOX2), a marker of stemness [15]. BIS depletion also inhibited GSC-like properties and SOX2 expression in glioblastoma cell lines under the same conditions [16]. However, little is known about the relationship between HSF1 and BIS in GSC-like SPs and the combined effect of BIS or HSF1 depletion and TMZ treatment on apoptosis. We hypothesized that BIS plays a key role in stemness-related properties via HSF1 regulation and that BIS or HSF1 depletion combined with TMZ treatment would induce apoptosis in SPs of the glioblastoma cell line A172.

## 2. Results

### 2.1. BIS Depletion Decreased Protein Levels of HSF1 as Well as Its Nuclear Localization in A172 Glioblastoma Cells under SP-Forming Conditions

**Figure 1:**
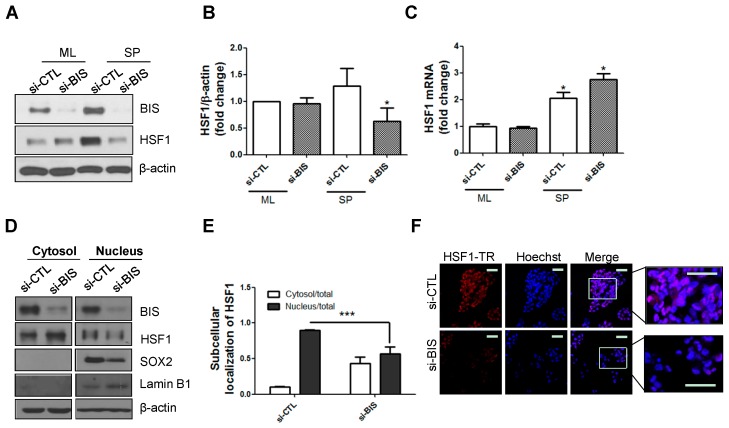
Bcl-2 interacting cell death suppressor (BIS) depletion decreased the protein, but not mRNA, levels of heat shock factor 1 (HSF1) in sphere (SP)-forming conditions. (**A**,**B**) Effect of BIS knockdown on HSF1 protein and (**C**) mRNA levels in A172 glioblastoma cells in standard monolayer (ML) and SP-forming culture conditions. Using small-interfering RNA for BIS (si-BIS), BIS expression was suppressed and then expression of HSF1 was analyzed by Western blotting and qRT-PCR. (* p < 0.05 vs. the siRNA control (si-CTL) treated group in ML); (**D**) Subcellular localization of HSF1 in the SP-forming condition was analyzed by subcellular fractionation and subsequent Western blotting; (**E**) Quantification of HSF1 levels in nuclear and cytosolic fraction was carried out with Image J software after normalization with Lamin B1 and β-actin for nuclear and cytosolic HSF1, respectively. The relative distribution of HSF1 after BIS depletion in nuclear and cytosolic fraction was determined from three independent experiments. *** *p* < 0.005 vs. si-CTL; (**F**) HSF1 was labeled with Texas Red (TR) in spheres of A172 cells and examined by confocal microscopy. Hoechst 33342 was utilized for nuclear staining. Scale bar: 50 μm.

We first confirmed the effect of BIS depletion on HSF1. A172 glioblastoma cells were transfected with 100 nM BIS small interfering RNA (siRNA) for 48 h and cultured under SP-forming conditions, which we established in a previous study [15]. As shown in Figure 1A,B, BIS depletion was verified via Western blotting compared with control siRNA (si-CTL) in both the standard monolayer (ML) and SP-forming culture conditions. Interestingly, protein expression of HSF1 was not altered in cells of the standard ML but was significantly downregulated in SPs following BIS depletion. To examine if the downregulated HSF1 protein level was due to mRNA level changes, we performed quantitative real-time polymerase chain reaction (qRT-PCR). As shown in Figure 1C, *HSF1* mRNA was slightly increased in SP-forming conditions and BIS depletion did not alter *HSF1* mRNA. We observed similar results in U87-MG glioblastoma cells (Figure S1A,B).

Next, we examined whether downregulation of BIS affected the subcellular localization of HSF1 in SPs with nuclear and cytosolic fractionation. Each fraction was verified with the nuclear and cytosolic markers Lamin B1 and β-actin, respectively. Consistent with our previous data, nuclear SOX2 was decreased with siBIS treatment. When BIS was knocked down with siRNA in A172 cells, nuclear translocation of HSF1 was significantly suppressed compared with control SPs (Figure 1D,E). Similarly, we observed decreased nuclear localization of HSF1 in BIS-depleted SPs via confocal microscopy (Figure 1F).

### 2.2. mRNA and Protein Levels of HSF1 Increased in SP-Forming Conditions and HSF1 Knockdown Inhibited SP Formation and Matrix Metalloprotease 2 (MMP2) Activity in A172 Cells

**Figure 2:**
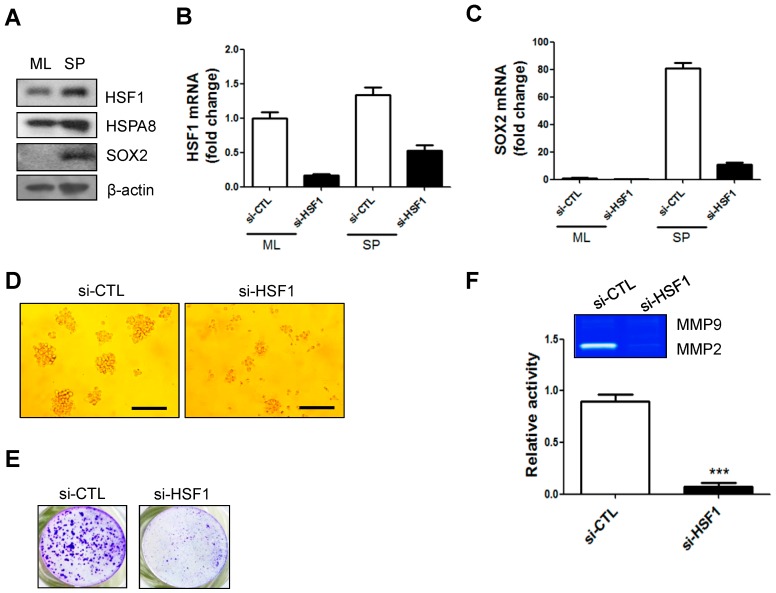
Protein and mRNA levels of HSF1 increased in SP-forming conditions and HSF1 depletion affected SP formation and MMP2 activity: (**A**) Western blot analysis of HSF1, HSPA8, and sex determining region Y (SRY)-box 2 (SOX2) in the ML and SP-forming conditions; (**B**,**C**) *HSF1* and *SOX2* mRNA levels in the ML or SP-forming conditions with or without HSF1 knockdown; (**D**,**E**) HSF1 depletion decreased SP formation. SPs were visualized by crystal violet staining. Scale bars: 200 μm, 200×; (**F**) Metalloprotease (MMP)2 activity was analyzed by gelatin zymography. *** *p* < 0.005 vs. siCTL.

Concomitant with increased HSF1 expression, we observed that BIS expression was also increased under SP-forming conditions [16]. Based on previous reports in which BIS was reported as a target gene of HSF1 [8,9,10], as well the data presented in Figure 1, we inquired whether the interaction between HSF1 and BIS may be involved in the regulation of glioblastoma stemness. We first investigated whether HSF1 was altered under SP-forming culture conditions, in which reactive oxygen species (ROS) as well as mRNA and protein expression of SOX2 are increased [15]. We compared HSF1 protein levels between the standard ML and SP-forming culture conditions for 72 h in A172 glioblastoma cells. Consistent with a previous report [15], SOX2 protein was increased in SP-forming conditions. Protein expression of HSF1, as well as its target gene *HSPA8*, was also increased in the SP-forming condition (Figure 2A). Following siRNA knockdown of HSF1 for 48 h, cells were transferred to ML or SP-forming culture conditions. We verified that HSF1 was depleted; qRT-PCR showed that *SOX2* mRNA was decreased in HSF1-depleted SPs (Figure 2B,C). Furthermore, sphere forming ability was suppressed by HSF1 depletion (Figure 2D,E). Since cancer stem cells were reported to display a high potential for epithelial–mesenchymal transition (EMT), which is subsequently linked to invasive phenotype, we checked matrix metalloprotease (MMP) activity as a critical player for EMT [17,18,19]. Zymography using supernatant from sphere incubation indicated HSF1 knockdown led to reductions in MMP2 activity, suggesting that HSF1 may play a role in the EMT which is might be associated with GSC-like phenotype (Figure 2D,F).

### 2.3. Transcription of HSF1 Target Genes Was Not Affected by Depletion of BIS or HSF1 in SP-Forming Conditions

**Figure 3:**
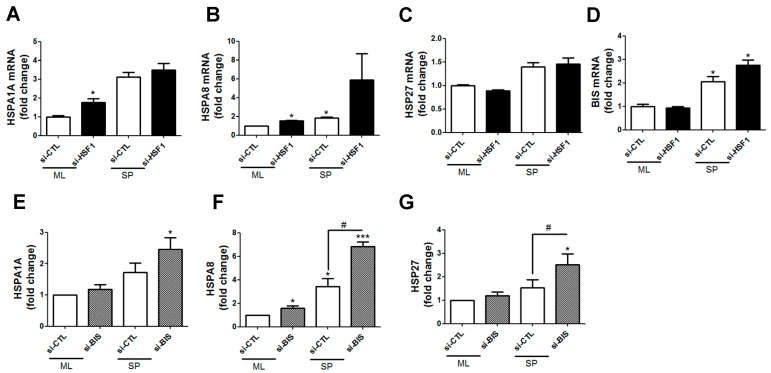
Effects of HSF1 (**A**–**D**) or BIS (**E**–**G**) depletion on transcription of HSF1 target genes. Quantitative real time polymerase chain reaction (qRT-PCR) analysis was performed in siRNA-treated ML and SP-forming conditions. * *p* < 0.05 and *** *p* < 0.005 vs. si-CTL in ML, # *p* < 0.05 vs. si-CTL in SP.

Next, we investigated whether representative *HSF1* target genes, such as *HSP*, were altered by siRNA depletion of HSF1 or BIS. qRT-PCR results indicated that *HSPs*, such as *HSPA8* and *HSP27*, were significantly upregulated with siHSF1 in SP-forming conditions (Figure 3A–D). Consistent with this, BIS depletion did not decrease transcription of *HSP* genes (Figure 3E–G). These results are similar to data obtained with the glioblastoma cell line U87 under the same conditions (Figure S1D–F).

### 2.4. SP A172 Cells Were Less Sensitive to TMZ Treatment Than Cells Cultured in the ML

**Figure 4:**
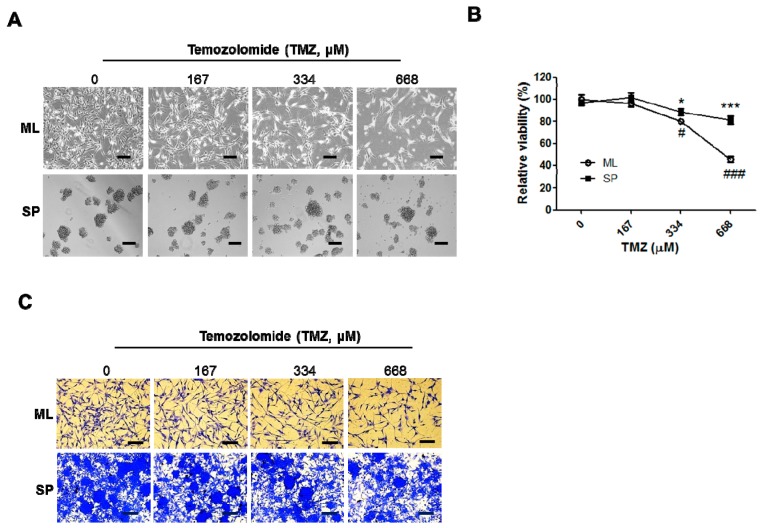
Temozolomide (TMZ)-induced cell death in the ML and SP-forming conditions. Following TMZ treatment for 48 h in the ML or SP-forming conditions, cellular images were taken with an inverted microscope (**A**) and the chemosensitivity was measured using a viability assay kit as described in Materials and Methods (**B**); (**C**) Crystal violet staining was performed after treatment of increasing dose of TMZ for A172 cells in ML and SP-forming conditions. Spheres were re-attached on l-ornithine-coated plates. * *p* < 0.05 and *** *p* < 0.005 vs. 0 μM TMZ-treated ML, # *p* < 0.05, ### *p* < 0.05 vs. 0 μM TMZ-treated SP. Scale bars: 200 μm.

TMZ is used for glioblastoma treatment, although resistance often develops. Hence, we investigated TMZ resistance of SPs expressing increased SOX2 compared with ML cells, which did not express SOX2. ML or SP cells were treated with increasing concentrations of TMZ (0, 167, 334, and 668 μM) for 48 h and chemosensitivity was measured using a viability assay kit to compare the cellular response to TMZ. As shown in Figure 4, A172 cells under SP condition were less sensitive to TMZ compared with the ML cells.

### 2.5. HSF1 Depletion Increased TMZ Sensitivity and Was Accompanied by a Decrease in SP Formation and SOX2 Expression

**Figure 5:**
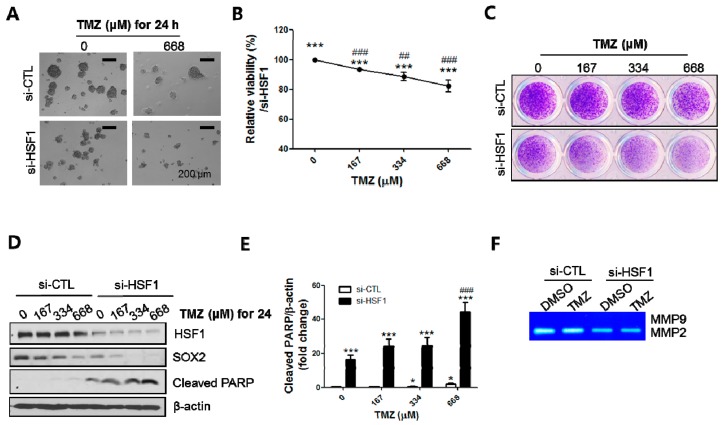
HSF1 depletion sensitizes TMZ-induced cell death. Following TMZ treatment on HSF1-depleted SPs, images were taken with an inverted microscope (**A**) and the relative viability was determined by mitochondrial reductase activity as described in Materials and Methods (**B**); (**C**) Crystal violet staining was performed after re-attachment of spheres on L-ornithine-coated plates; (**D**,**E**) Western blotting assays for HSF1, SOX2 and cleaved PARP were performed and the levels of cleaved PARP were determined by quantitation of intensities of bands in Western blots by Image J software from three independent experiments; (**F**) Zymography for MMP2 was performed following treatment of TMZ (668 µM) with control or HSF siRNA. * *p* < 0.05, *** *p* < 0.005 vs. 0 μM of TMZ treated with si-CTL. ### *p* < 0.005 vs. 0 μM of TMZ treated with si-HSF1. Scale bars: 200 μm.

The results presented in Figure 2 and Figure 4 suggested that high levels of HSF1 may be important in TMZ resistance, raising the possibility that downregulation of HSF1 may sensitize the response to TMZ. To investigate this, we pretreated cells with 50 nM siHSF1 for 48 h, trypsinized, and transferred the cells to SP-forming culture conditions. The SPs were then exposed to different concentrations of TMZ (0, 167, 334 and 668 μM). After 24 to 48 h of treatment, SPs were imaged with an inverted microscope and analyzed with a viability assay, SP-formation assay, and Western blotting. HSF1 depletion suppressed SP formation and viability upon TMZ treatment (Figure 5A–C). SOX2 expression was decreased by TMZ in a concentration dependent manner, which was further decreased by HSF1 depletion (Figure 5D). HSF1 silencing also enhanced the cleavage of poly (ADP-ribose) polymerase (PARP), a marker of apoptosis, compared with TMZ treatment only (Figure 5D,E). Next, supernatant from the SP media was analyzed with zymography. We found that HSF1 depletion inhibited MMP2 activity; however, the inhibition was not increased when HSF1 depletion was combined with TMZ treatment (Figure 5F). Consistent with this, treatment with BIS siRNA and TMZ also significantly induced apoptosis concomitantly with suppression of CSC-like properties (Figure S2). Therefore, these results indicate that BIS and HSF1 depletion enhances the sensitivity to TMZ treatment in GSCs.

## 3. Discussion

HSF1 is considered not only a master regulator of HSP induction at the transcriptional level, but also as a powerful modifier of carcinogenesis by enhancing cellular proliferation and survival in response to oncogenic stimuli [20]. HSF1 has also been shown to be involved in hepatocellular carcinoma development [3], as well as induction of a CSC-like phenotype in breast cancer cells [5]. A greater understanding of CSCs will be important for the diagnosis and treatment of cancer as well as for future drug development [21,22].

BIS is a target gene of HSF1 and is regulated by HSF1 under conditions of stress. In addition to the transcriptional regulation of BIS by HSF1 [8], BIS and HSF1 physically interact with one another [12,14]. Recently, Gentilella et al. reported that ectopic expression of BIS resulted in remarkable increase in the level of HSF1 in nuclear fraction in T98G and U87 glioblastoma cells, indicating the regulatory role for BIS in the activation of HSF1 in glioblastoma cells [23]. However, little is known regarding the role of BIS and HSF1 in GSC-like SPs. A previous study demonstrating that BIS was involved in regulation of the GSC-like phenotype via signal transducer and activator of transcription 3 (STAT3) stabilization prompted us to investigate whether BIS depletion might affect HSF1 in SP-forming conditions [16]. In the present study, we demonstrated that BIS depletion downregulated HSF1 protein levels and altered its subcellular localization. TMZ treatment in conjunction with BIS or HSF1 depletion increased apoptosis and inhibited the GSC-like phenotype. To the best of our knowledge, this is the first evidence of BIS-mediated HSF1 regulation in the GSC-like phenotype. Jin et al. recently identified a role for BIS in the regulation of nuclear shuttling of HSF1 during heat stress (HS) [13], consistent with our finding that BIS depletion reduced HSF1 nuclear localization under SP-forming conditions, in which ROS generation and SOX2 expression are increased [17].

It is well known that GSCs are resistant to anti-cancer drugs, such as TMZ. For instance, Garros-Regulez et al. found that TMZ resistance was positively correlated with SOX2 expression in glioblastoma cells and in cells expressing high levels of SOX2 [24]. When we tested this in our culture system, we also consistently observed that SPs expressing SOX2 [15,25] were more resistant to TMZ treatment compared with ML cells, suggesting that our culture system is suitable for in vitro study of GSCs and the drug response [26]. Based on this, we examined the effect of BIS or HSF1 depletion in combination with TMZ treatment, and found that apoptosis was increased (as measured by PARP cleavage), SOX2 levels were decreased, and SP formation was increased. However, it should be noted that HSF1 silencing itself resulted in the considerable decrease in SOX2 levels as well as increase in PARP cleavage. Even though the effect of HSF1 depletion on the TMZ sensitivity is not synergistic but only additive, our results provide the possibility that targeting HSF1 could be used as an adjunct to conventional TMZ treatment for glioblastoma to diminish the dose of TMZ as well as its toxic side effects.

TMZ resistance has been linked to increased expression of O6-methylguanine DNA methyltransferase (MGMT) [27,28,29]. To investigate whether BIS-mediated HSF1 downregulation had an effect on MGMT, we analyzed *MGMT* expression using qRT-PCR in A172 and U87 cells. We were not able to detect mRNA expression of MGMT in A172 and U87 cells, suggesting that the inhibitory effect of BIS-mediated HSF1 downregulation on the GSC-like phenotype is MGMT-independent (Figure S3). It has been reported that BIS expression stabilizes myeloid leukemia cell differentiation protein (MCL)-1 and promotes cellular survival in a neuroblastoma cell line [30]. In contrast, cisplatin treatment decreased MCL-1 protein levels, leading to increased cell death in BIS-depleted A549 cells [31]. Hence, it is possible that BIS depletion may affect MCL-1 stabilization, thereby enhancing TMZ-induced apoptosis.

Although the underlying mechanism explaining how BIS downregulates HSF1 in SP-forming conditions is unknown, it is possible that BIS regulates HSF1 post-transcriptionally through ubiquitin-mediated protein degradation or autophagy since *HSF1* mRNA was increased by BIS depletion. Previous studies, as well as increasing evidence, support this postulation as BIS has been shown to regulate several proteins, including microtubule-associated protein 1 light chain 3β (MAP1LC3B) [32], STAT3 [16], and myosin heavy chain [33].

We also unexpectedly observed that HSF1 depletion did not suppress representative target genes of HSF1 (*HSPA8*, *HSPA1A*, *HSP27*, and *BIS*) in the SP-forming condition, suggesting that unknown transcription factors might regulate *HSPs* independent of HSF1. There have been reports of HSF1-independent regulation of HSPs. Mendillo et al. analyzed a HSF1-regulated transcriptional program specific to highly malignant cells and found that it was distinct from HS, which induced representative HSF1-target genes, such as *HSPA1A* [34]. Wang et al. also reported that HSF1 knockdown or overexpression did not significantly alter the expression of *HSPA1A* or *HSP90*, suggesting that non-*HSP* targets of HSF1 may be involved in the regulation of the CSC-like phenotype [5]. Hence, there could be alternative pathways for HSP transcription. For example, human HSF2, but not HSF1, homotrimerizes and functionally complements the viability defect associated with deletion of the yeast *HSF* gene [35]. In another study, Lang et al. demonstrated that HS induces epithelial plasticity and cell migration independent of HSF1 [36]. This group showed that HSF1 knockdown in the A549 model did not prevent the morphological changes or enhanced migratory profile of heat-stressed cells, suggesting that HS significantly impacts cancer cell epithelial plasticity and the migratory phenotype independently of HSF1. Moreover, Matsuda et al. reported that nestin regulates stemness, cell growth, and invasion in glioblastoma cells by altering *HSPA8* [37]. Those reports support our findings that *HSPs* could be regulated by an HSF1-independent mechanism.

## 4. Materials and Methods

### 4.1. Cell Culture and Small Interfering RNA (siRNA) Transfection

A172 and U87 human glioblastoma cells, A549 human lung cancer cells and HEP2 human laryngeal cancer cells were purchased from American Type Culture Collection (ATCC, Manassas, VA, USA) and maintained in DMEM (Hyclone, Logan, UT, USA) contained with 10% heat-inactivated fetal bovine serum (FBS), 100 units/mL penicillin and 100 mg/mL streptomycin at 37 °C in 5% CO_2_ atmosphere. For the sphere-forming assay, a single-cell suspension following trypsinization was cultured in B27-supplemented DMEM/F12 (Cellgro, Manassas, VA, USA) with epidermal growth factor and basic fibroblast growth factor (10 ng/mL each: R&D Systems, Minneapolis, MN, USA) without serum on ultralow attachment plates (Corning, Tewksbury, MA, USA) at a density of 1 × 10^5^ cells/mL as described previously [17]. Knockdown was performed by transfection of specific 100 nM siRNA G-fectin (Genolution Pharmaceuticals, Seoul, Korea) according to manufacturer’s instruction. siRNAs for control (si-CTL: 5′-CCUACGCCACCAAUUUCGU-3′), and BIS (si-BIS: 5′-AAGGUUCAGACCAUCUUGGAA-3′) were purchased from Bioneer (Daejeon, Korea). Specific siRNA targeted for HSF1 (si-HSF1: 5′-CUGAAGAGUGAAGACAUAAAGA-3′) was purchased from Genolution Pharmaceuticals. Temozolomide was purchased from Sigma-Aldrich (St. Louis, MO, USA) and dissolved in dimethyl sulfoxide (Sigma-Aldrich).

### 4.2. Sphere-Formation Assay

Spheres after 3–5 days were attached to standard culture plates in media containing 5% FBS stained with crystal violet solution (Sigma-Aldrich, St. Louis, MO, USA). For morphological examination, pictures were taken under the inverted microscope.

### 4.3. Western Blot

Cells were lysed with lysis buffer (150 mM NaCl, 1% NP-40, 0.5% sodium deoxycholate, 0.1% SDS, 50 mM Tris–HCl pH 8.0) with protease inhibitor (Roche Diagnostics, Mannheim, Germany) on ice for 30 min. Equal amounts of protein were separated on 10% sodium dodecyl sulfate polyacrylamide gel electrophoresis (SDS-PAGE) and transferred to nitrocellulose membranes (GE Healthcare Life Sciences, Buckinghamshire, UK). The membranes were incubated for 1 h with 5% dry skim milk in TBST (20 mM Tris, 137 mM NaCl, 0.1% Tween 20) buffer and then incubated with antibodies against BIS [6], HSF1, HSPA8 (Enzo Life Sciences, Farmingdale, NY, USA), SOX-2 (Santa Cruz Biotechnology, Santa Cruz, CA, USA), cleaved PARP (Abcam, Cambridge, UK) and β-actin (Sigma-Aldrich). After incubation with horseradish peroxidase-conjugated anti-mouse, anti-rabbit or anti-goat IgG (1:5000; Santa Cruz Biotechnology), the immunoreactive bands were visualized by an enhanced chemiluminescence substrate (Thermo Fisher Scientific, Waltham, MA, USA). Quantification for the intensities of each band was carried out on Image J software (NIH, Bethesda, MD, USA).

### 4.4. Subcellular Fractionation

Cells were incubated in buffer A (10 mM HEPES, 1.5 mM MgCl_2_, 10 mM KCl, 0.5 mM dithiothreitol (DTT) and 0.05% NP40, pH 7.9) for 10 min on ice. After centrifugation at 3000 rpm for 10 min, supernatant was utilized as a cytosolic fraction. Pellet was suspended in buffer B (5 mM HEPES, 1.5 mM MgCl_2_, 0.2 mM EDTA, 0.5 mM DTT, 300 mM NaCl and 26% glycerol, pH 7.9), homogenized 20 times with a plastic pestle homogenizer, and incubated on ice for 30 min. After centrifugation at 13,200 rpm for 30 min, supernatant was used as a nuclear fraction. Lamin B1 (Santa Cruz Biotechnology) was utilized as a nuclear marker protein.

### 4.5. Confocal Microscopy

Spheres attached on poly-l-ornithine-treated plates were fixed for 10 min with 3.7% formaldehyde in phosphate buffered saline (PBS) and permeabilized for 10 min at room temperature with 0.2% Triton X-100. The spheres were blocked for 1 h with 1% bovine serum albumin in PBS at room temperature. Specific primary antibody for HSF1 was added to the cells, and incubated overnight at 4 °C. The cells were washed in PBS and incubated for 1 h at 4 °C with fluorescent Texas Red—conjugated anti rabbit IgG (Santa Cruz Biotechnology). After washing, the cells were stained with 0.4 mg/mL 40, 6-diamidino-2-phenylindole (DAPI, Sigma) for 10 min at room temperature. Fluorescent images were acquired via confocal microscopy (Zeiss, Oberkochen, Germany).

### 4.6. Quantitative Real-Time Polymerase Chain Reaction (qRT-PCR)

Total RNA was isolated using AccuZol (Bioneer), and first strand of cDNA was synthesized by reverse transcribed to cDNA using a ReverTra Ace qPCR kit (Toyobo, Osaka, Japan). A quantitative real-time PCR was performed to validate the expression level using SYBR^®^ Premix Ex TaqTM (TaKaRa Bio, Shiga, Japan) with specific primers (Table 1) on Applied Biosystems 7300 machine (Applied Biosystems, Carlsbad, CA, USA). The relative values for specific mRNA were calculated after normalization to the *C*t value from β-actin in the same sample using the DDCt method.

### 4.7. Gelatin Zymography

Gelatin zymography was performed using supernatants from sphere forming culture media as previously described [38].

### 4.8. Cell Viability Assay

Cell proliferation was assessed as a function of metabolic activity using an EZ-Cytox Cell Viability Assay kit (ItsBio, Seoul, Korea) as mentioned in the previous study [17]. EZ-Cytox Cell Viability assay is based on reduction of tetrazolium chloride to the water-soluble formazan by succinate-tetrazolium reductase, which forms part of the mitochondrial respiratory chain.

### 4.9. Statistics

The Student *t*-test was used to compare the differences between two groups. Each experiment was repeated at least three times and *p*-values of <0.05 were considered as statistically significant.

## 5. Conclusions

In summary, the present study demonstrates that BIS depletion downregulates HSF1 protein levels under SP-forming conditions and the combination of TMZ treatment and downregulation of BIS or HSF1 significantly inhibits the GSC-like phenotype and increases apoptosis. Our results suggest that BIS or HSF1 could be a therapeutic target for GSCs resistant to conventional TMZ treatment.

## Figures and Tables

**Table 1:** Primers for qRT-PCR.

Gene	Sequence (5'-3')
BIS	Forward: 5′-GGAATTCGCATGAGCGCCGCCACCCACTCG-3′
	Reverse: 5′-CTCGAGCTACGGTGCTGCTGGGTTACCAGG-3′
SOX2	Forward: 5′-TACCTCTTCCTCCCACTCCA-3′
	Reverse: 5′-ACTCTCCTCTTTTGCACCCC-3′
HSF1	Forward: 5′-GGCCATGAAGCATGAGAATG -3′
	Reverse: 5′-GTTCAGCATCAGGGGGATCT-3′
HSPA1A	Forward: 5′-AGCTGGAGCAGGTGTGTAAC-3′
	Reverse: 5′-CAGCAATCTTGGAAAGGCCC-3′
HSPA8	Forward: 5′-TAACCCCATCATCAGCGGAC-3′
	Reverse: 5′-TCCCAACAGTCCACCTCAAAG-3′
HSP27	Forward: 5′-TGACGGTCAAGACCAAGGAT-3′
	Reverse: 5′-ATGGTGATCTCGTTGGACTG-3′
MGMT	Forward: 5′-CCGTTTGCGACTTGGTACTT-3′
	Reverse: 5′-CCTTGCCCAGGAGCTTTATTT-3′
β-ACTIN	Forward: 5′-AGTACTCCGTGTGGATCGGC-3′
	Reverse: 5′-GCTGATCCACATCTGCTGGA-3′

## Supplementary Materials

Supplementary materials can be found at www.mdpi.com/1422-0067/18/2/468/s1.

## Abbreviations

BIS	Bcl-2 (B-cell lymphoma 2) interacting cell death suppressor
CSC	Cancer stem cell
DTT	Dithiothreitol
HSEs	Heat shock elements
HSF1	Heat shock factor 1
HSPs	Heat shock proteins
GBM	Glioblastoma multiforme
GSCs	Glioblastoma stem cells
si-CTL	control siRNA
siRNA	Small interfering RNA
SP	Sphere
MMP2	Matrix metalloprotease 2
PARP	Poly(ADP-ribose) polymerase
SOX2	Sex determining region Y (SRY)-box 2
TMZ	Temozolomide
MAP1LC3B	Microtubule-associated protein 1 light chain 3 beta
MCL-1	Myeloid leukemia cell differentiation protein
MGMT	O6-methylguanine DNA methyltransferase
ML	Monolayer
qRT-PCR	Quantitative real-time polymerase chain reaction
ROS	Reactive oxygen species
STAT3	Signal transducer and activator of transcription 3
