# Supplementary material for: Heat Shock Factor 1 Depletion Sensitizes A172 Glioblastoma Cells to Temozolomide via Suppression of Cancer Stem Cell-Like Properties
Source: Int J Mol Sci. 2017 Feb 22;18(2):468. doi: 10.3390/ijms18020468 (PMC5344000; doi:10.3390/ijms18020468)
Supplement: Supplementary file 1 [file ijms-18-00468-s001.pdf]

Supplementary Materials: Heat Shock Factor 1 Depletion Sensitizes A172 Glioblastoma Cells to Temozolomide via Suppression of Cancer Stem Cell-Like Properties

Chang-Nim Im, Hye Hyeon Yun and Jeong-Hwa Lee

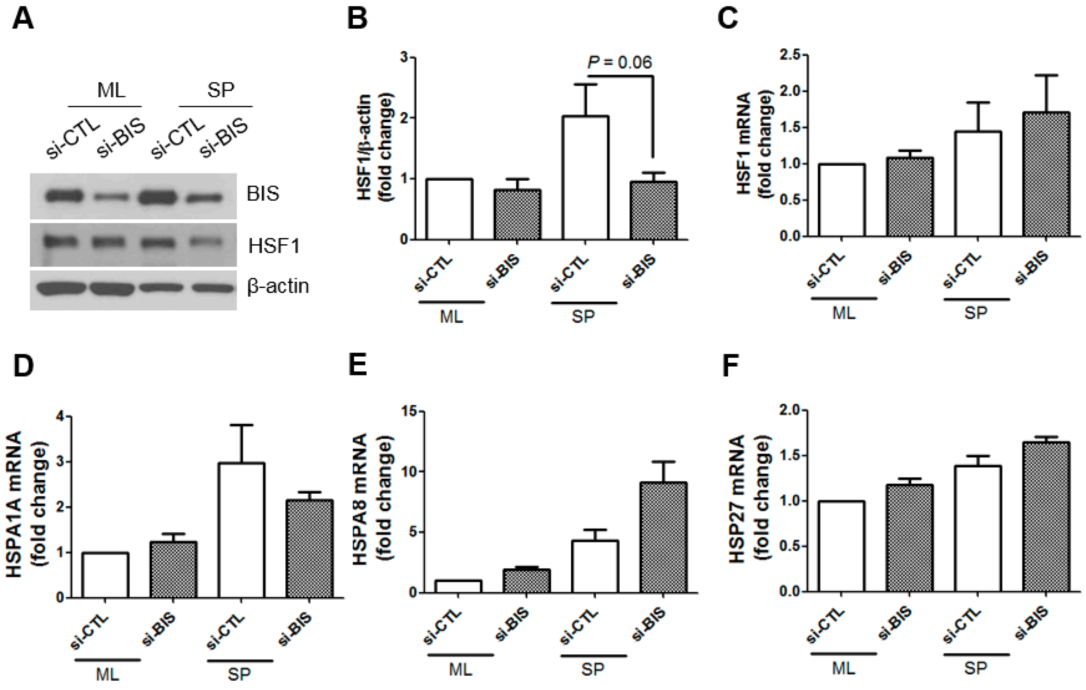

Figure S1. BIS depletion decreases protein, but not mRNA, levels of heat shock factor 1 (HSF1) in U87 cells under sphere (SP)-forming conditions. (**A**); (**B**) The effect of BIS knockdown on HSF1 protein and (**C**) mRNA in U87-MG cells in the monolayer (ML) and SP-forming culture conditions.

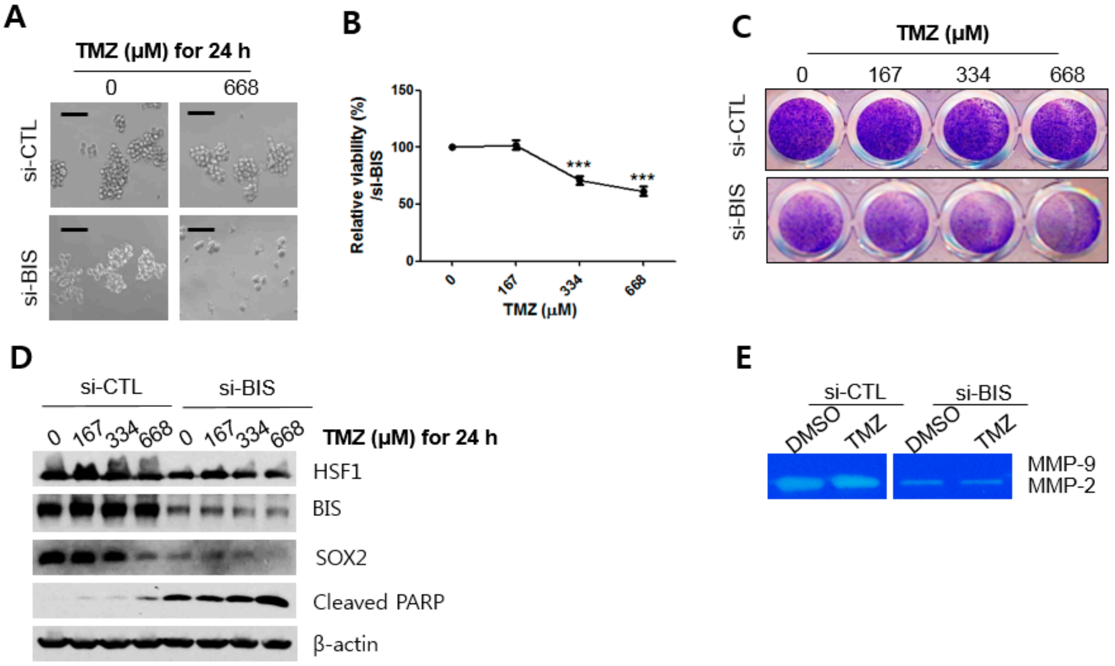

Figure S2. BIS depletion sensitizes temozolomide (TMZ)-induced cell death in A172 glioblastoma cell death in SPs. Following treatment of small interfering BIS (si-BIS), SP of A172 glioblastoma were incubated with TMZ, images were taken with an inverted microscope (**A**) and viability was determined (**B**); (**C**–**E**) Crystal violet staining, Western blotting and zymography were performed as described in the Materials and Methods. Scale bars: 100 μm.

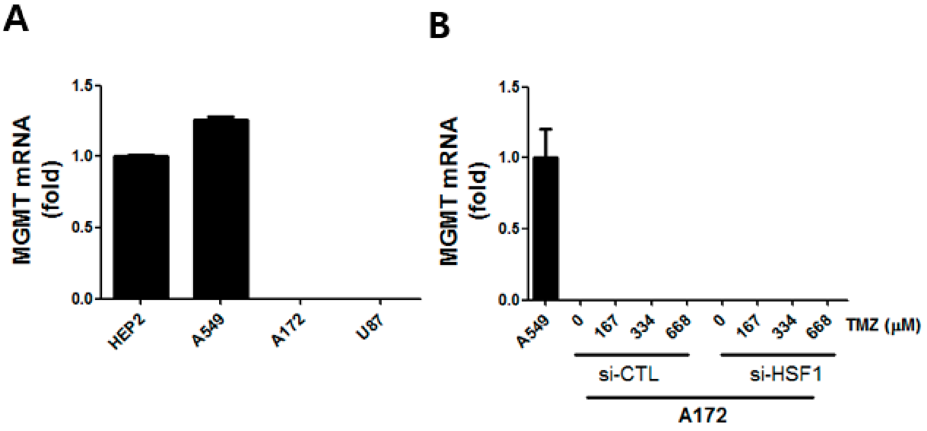

Figure S3. MGMT mRNA levels in several cancer cell lines. (**A**) MGMT mRNA was not detected in A172 and U87 cells. (**B**) The MGMT mRNA levels in HEP2 cells were arbitrarily designated as 1.0; (**B**) Both HSF1 depletion and TMZ treatment did not affect MGMT mRNA levels in A172 cells. The MGMT mRNA levels in A549 cells were arbitrarily designated as 1.0.
